# Local Insulin for Local Needs? Insights into Retinal Insulin Signaling and RPE Metabolism

**DOI:** 10.3390/biom15111570

**Published:** 2025-11-08

**Authors:** Matilde Balbi, Alessandra Puddu, Andrea Amaroli, Davide Maggi, Isabella Panfoli, Silvia Ravera

**Affiliations:** 1 Department of Experimental Medicine, University of Genoa, Via De Toni 14, 16132 Genova, Italy; matilde.balbi@unige.it (M.B.); silvia.ravera@unige.it (S.R.); 2 Department of Internal Medicine and Medical Specialties, University of Genova, Viale Benedetto XV 6, 16132 Genova, Italy; apuddu@unige.it (A.P.); davide.maggi@unige.it (D.M.); 3 BIO-Photonics Overarching Research Laboratory (BIOPHOR), Department of Earth, Environmental and Life Sciences (DISTAV), University of Genoa, 16132 Genoa, Italy; andrea.amaroli@unige.it; 4 Department of Pharmacy—(DIFAR), University of Genoa, Viale Benedetto XV 3, 16132 Genova, Italy; 5 IRCCS Ospedale Policlinico San Martino, Largo Rosanna Benzi 10, 16132 Genova, Italy

**Keywords:** insulin, retinal pigment epithelium, photoreceptors, retinal diseases, glucose metabolism, glucose uptake

## Abstract

Insulin is a key anabolic hormone traditionally considered to be exclusively produced by pancreatic β-cells. Insulin exerts several systemic effects involved in glucose uptake and metabolism. In the retina, insulin signaling acts as a regulator of photoreceptor- retinal pigment epithelium (RPE) metabolic coupling as well as of neuronal survival via the PI3K/Akt and MAPK/ERK pathways. Impaired insulin signaling contributes to diabetic retinopathy, retinitis pigmentosa, and age-related degeneration by disrupting energy homeostasis and trophic support. However, growing evidence suggests that the retina, particularly RPE, locally synthesizes and secretes insulin. Although the role of local insulin production in the retina remains to be clarified, this discovery introduces a paradigm shift in retinal physiology, suggesting a self-sustaining insulin signaling system that supports glucose uptake, lipid metabolism, and neurovascular integrity. Emerging data indicate that RPE-derived insulin is stimulated by photoreceptor outer segment (POS) phagocytosis and may act through autocrine and paracrine mechanisms to maintain retinal function, even under conditions of systemic insulin deficiency. Understanding this extra-pancreatic insulin source opens new therapeutic perspectives aimed at enhancing local insulin signaling to preserve vision and prevent retinal degeneration. Thus, the objective of this review is to summarize current evidence on RPE-derived insulin and to discuss its potential implications for retinal homeostasis and disease.

## 1. Introduction

Although functions of pancreatic insulin are well characterized, evidence of local insulin production within the retina, particularly by the retinal pigment epithelium (RPE) [1,2], offers new perspectives on retinal metabolism and neurovascular regulation. However, the regulation and functional consequences of this locally produced insulin remain poorly understood.

Therefore, the objective of this review is to summarize the role of pancreatic insulin (also named “systemic insulin”) on retina metabolism and proliferation, as well as to discuss the current findings on RPE-derived insulin (also named “local insulin”) and its potential physiological and pathological roles.

## 2. Insulin and Its Pancreatic Production: Molecular Mechanisms and Physiological and Pathological Implications

Insulin is a pivotal anabolic hormone that regulates systemic glucose and energy homeostasis [3,4]. It is synthesized by pancreatic β-cells belonging to the islets of Langerhans in response to rising blood glucose levels [5]. Insulin regulates the glucose uptake, storage, and utilization, favoring glucose uptake in insulin-sensitive tissues such as skeletal muscle and adipose tissue [3,6,7]. In addition, insulin suppresses gluconeogenesis and glycogenolysis [8,9] and stimulates glycogen synthesis and lipogenesis in the liver to promote energy storage [9,10].

Beyond glucose metabolism, insulin exerts important effects on protein and lipid turnover since it enhances amino acid uptake and protein synthesis while inhibiting proteolysis, and promotes lipid synthesis while limiting lipolysis and ketogenesis [11,12].

Insulin also plays a role in vascular function [13], cell growth [14], and gene expression [15], reflecting its pleiotropic nature.

Dysregulation of insulin secretion or signaling results in systemic disorders driven not only by defective glucose uptake as in diabetes, but also by secondary microvascular and macrovascular damage, including, among others, retinal diseases [16,17,18,19].

### 2.1. Pancreatic Insulin Production and Release

**Figure 1:**
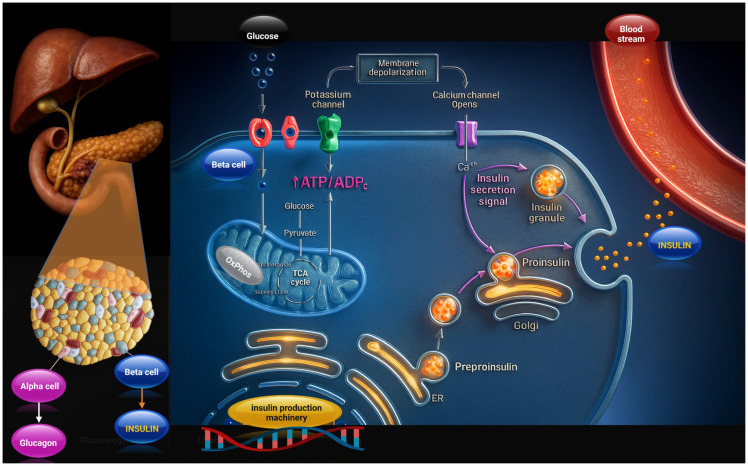
Schematic representation of pancreatic β-cell insulin biosynthesis and secretion. Glucose uptake increases the ATP/ADP ratio, leading to potassium channel closure, membrane depolarization, calcium influx, and insulin granule exocytosis into the bloodstream. The image was created at https://BioRender.com by A.A (accessed on 28 October 2025)..

Pancreatic insulin production is carried out by the islets of Langerhans, clusters of endocrine cells, representing about 1–2% of the total pancreatic mass [20] (Figure 1). Among the different islet cell types, β-cells are the most abundant and are responsible for insulin production, working in close coordination with glucagon-secreting α-cells to regulate blood glucose levels [20]. β-cells are strategically positioned to sense fluctuations in circulating glucose, allowing for rapid insulin secretion [21]. Loss or dysfunction of these cells is a key feature of diabetes mellitus, making the islets of Langerhans an important focus of therapeutic research, including islet transplantation and stem-cell-derived β-cell replacement approaches [22].

Insulin biosynthesis begins with transcription of the *INS* gene and translation of preproinsulin, which is directed into the endoplasmic reticulum (ER), where the signal peptide is removed, producing proinsulin. Within the ER, proinsulin folds into its proper conformation thanks to chaperonin-mediated formation of three disulfide bonds. Misfolded molecules are eliminated through ER-associated degradation, ensuring protein quality control. Properly folded proinsulin is transported to the Golgi apparatus and packaged into secretory granules, where prohormone convertases PC1/3 and carboxypeptidase E process it into mature insulin and C-peptide. The granules’ acidic environment and high zinc concentration promote insulin crystallization and storage [23,24].

Blood glucose concentration represents the principal physiological stimulus for insulin secretion [25,26]. It enters β-cells through GLUT2 transporters in rodents (GLUT1/3 in humans) [27], where it is phosphorylated by glucokinase and metabolized, increasing the ATP/ADP ratio. This leads to closure of ATP-sensitive potassium channels, which causes, in turn, membrane depolarization and opening of voltage-gated calcium channels. The resulting calcium influx triggers exocytosis of insulin-containing granules through the coordinated action of SNARE proteins and synaptotagmin [28,29].

Insulin secretion is also stimulated by other nutrients and hormones. For example, amino acids such as leucine and arginine enhance insulin release by stimulating mitochondrial metabolism or depolarizing the plasma membrane, while free fatty acids act through G-protein-coupled receptors like GPR40/FFAR1 [30,31].

Regarding hormone signal, glucagon-like peptide-1 (GLP-1) and glucose-dependent insulinotropic polypeptide (GIP), both belonging to the incretin family, amplify glucose-stimulated insulin secretion by activating cAMP-dependent pathways involving protein kinase A (PKA) and Epac2, which promote granule priming and exocytosis [32,33]. In addition, incretins enhance calcium signaling and exert protective and proliferative effects on β-cells, reinforcing their secretory capacity in a glucose-dependent manner [34,35].

Also, neurotransmitters and stress-related hormones contribute to insulin release. Acetylcholine stimulates insulin secretion via muscarinic M3 receptors, while sympathetic activation through α_2_-adrenergic receptors inhibits it [36,37]. Glucagon and β-adrenergic signaling can enhance insulin output through cAMP-mediated pathways [38], whereas prolonged exposure to cortisol or catecholamines modulates β-cell function in response to metabolic stress [39].

In the complex, these regulatory networks ensure that insulin production and secretion match the body’s metabolic demands.

### 2.2. Molecular Mechanisms of Insulin Action

Insulin acts via the insulin receptor (IR), widely expressed in target tissues, including liver, muscle, adipose tissue, and endothelium [40]. IR is a heterotetrameric transmembrane tyrosine kinase formed by two extracellular α-subunits and two transmembrane β-subunits, which undergo autophosphorylation upon ligand binding [41]. When insulin binds to the α-subunits, conformational changes activate the intrinsic kinase activity of the β-subunits, leading to autophosphorylation of specific tyrosine residues [42]. These phosphorylated motifs serve as docking sites for adaptor proteins such as insulin receptor substrates (IRS), which are subsequently phosphorylated on tyrosine residues and act as scaffolds for downstream signaling [43,44].

The phosphoinositide 3-kinase (PI3K)/Akt cascade is the principal pathway among the signals activated by the IR [45,46]. In detail, the activation of PI3K catalyzes the conversion of Phosphatidylinositol phosphates (PIP)2 to PIP3 at the plasma membrane, recruiting phosphoinositide-dependent kinase-1 (PDK1) and Akt (protein kinase B). Activated Akt regulates multiple metabolic processes, including GLUT4 translocation to the cell surface, glycogen synthesis via inhibition of glycogen synthase kinase-3 (GSK3), suppression of gluconeogenesis through modulation of Forkhead box O (FOXO) transcription factors [45,47], and stimulation of protein synthesis through mTORC1 signaling [48] and lipogenesis through SREBP-1c-dependent transcription of fatty acid synthase and acetyl-CoA carboxylase [49]. At the same time, insulin signal inhibits lipolysis by activating phosphodiesterase (PDE3B), which breaks down cyclic AMP (cAMP), and by stimulating the dephosphorylation of the enzyme hormone-sensitive lipase [50].

IR also activates the Ras/MAPK (mitogen-activated protein kinase) pathway through recruitment of the adaptor protein Grb2 and the guanine nucleotide exchange factor SOS, leading to Ras activation and a kinase cascade involving Raf, MEK, and ERK. This pathway contributes to cell growth, differentiation, and gene expression [51].

The regulation of insulin signaling is ensured by negative regulators such as protein tyrosine phosphatases (e.g., PTP1B), suppressor of cytokine signaling proteins, and serine/threonine phosphorylation of IRS proteins, which can impair signal propagation [52].

Beyond classical metabolic effects, insulin acts on the vasculature via PI3K/Akt-mediated activation of endothelial nitric oxide synthase, improving nutrient and hormone delivery [53]. Conversely, impaired insulin signaling leads to endothelial dysfunction, increased oxidative stress, and inflammation, which contribute to the pathogenesis of diabetic microvascular complications such as retinopathy, nephropathy, and neuropathy [54,55,56]. Similarly, insulin signaling in the central and peripheral nervous system supports neuronal survival, synaptic plasticity, and energy metabolism [57]. Additionally, via the MAPK/ERK pathway, insulin mediates mitogenic effects on growth and gene expression [15].

### 2.3. Insulin Systemic Metabolic Effects

Since insulin is a central regulator of systemic metabolism, it acts on several tissues involved in energy homeostasis maintenance (Table 1). Among these, liver, skeletal muscle, and adipocytes represent the major sites for nutrient uptake, storage, and mobilization [58].

Skeletal muscle represents the largest site of postprandial glucose disposal, playing a primary role in systemic insulin sensitivity [59,60]. In this tissue, insulin promotes GLUT4 translocation to the plasma, enhancing glucose uptake [47], and stimulates protein synthesis and inhibits proteolysis, supporting muscle anabolism [48].

In adipose tissue, insulin promotes GLUT4-mediated glucose uptake [61] and favors lipogenesis [49], facilitating triglyceride storage by increasing glycerol-3-phosphate availability, and inhibits lipolysis [50].

Glucose and lipid metabolism are also coordinated by the liver, which integrates hormonal and nutrient signals [10]. In this context, insulin signal suppresses hepatic gluconeogenesis, reducing expression of key enzymes including phosphoenolpyruvate carboxykinase and glucose-6-phosphatase [9]. Simultaneously, it promotes glycogen synthesis and stimulates de novo lipogenesis, increasing fatty acid and triglyceride production [62]. Insulin also regulates hepatic protein metabolism by enhancing amino acid uptake and translation [63].

The crosstalk among liver, muscle, and adipose tissue, mediated by hormones, cytokines, and metabolites, forms a tightly regulated network essential for systemic metabolic homeostasis. For example, hepatokines released by the liver modulate muscle insulin sensitivity, myokines secreted during exercise enhance hepatic glucose metabolism and adipose tissue function [64], and adipokines such as adiponectin improve insulin action in both liver and skeletal muscle [65].

When the hormone interconnection between these three organs does not work, as observed in insulin resistance, glucose, lipid, and protein metabolisms are altered, leading to hyperglycemia, dyslipidemia, and increased cardiovascular risk, hallmarks of type 2 diabetes and metabolic syndrome [66,67,68].

**Table 1:** Comparison of Insulin Receptor Expression in the Main Insulin-target Tissues and Major Downstream Reactions. Notes: Receptor expression and pathway dominance vary by tissue, species, and metabolic state. PI3K/Akt is the primary metabolic pathway mediating glucose uptake, glycogen/lipid synthesis, and eNOS activation. MAPK/ERK mainly regulates proliferation and differentiation.

Cell Type	Insulin Receptor (IR) Relative Expression/Function	Major Downstream Pathways Activated	Key Metabolic and Functional Outcomes	References
Pancreatic β-cell	Moderate IR expression; autocrine/paracrine roles—IR influences β-cell function and survival.	PI3K → Akt (survival, proliferation, transcriptional effects) and MAPK/ERK (growth/secretion modulation).	Regulation of insulin secretion (modulatory/autocrine), β-cell growth and survival, gene expression related to secretory machinery.	[69,70]
Skeletal muscle (myocytes)	High functional IR at the surface of insulin-responsive fibers; key peripheral glucose sink.	PI3K → Akt → AS160/TBC1D4 cascade and also MAPK/ERK. The PI3K pathway is required for GLUT4 translocation.	Rapid GLUT4 translocation → ↑ glucose uptake; glycogen synthesis (via GSK3 inhibition); protein synthesis (mTOR).	[71,72]
Adipocyte (white adipose tissue)	High IR expression; insulin controls adipocyte differentiation and lipogenesis.	PI3K → Akt → mTORC1 and SREBP-1c induction; MAPK/ERK involved in proliferation/differentiation.	↑ GLUT4 translocation and glucose uptake, ↑ lipogenesis (SREBP-1c → lipogenic genes), inhibition of lipolysis (via PDE3B/cAMP pathways).	[73,74]
Hepatocyte (liver)	High expression of IR; the liver is a major insulin target.	PI3K → Akt (dominant) and MAPK/ERK. PI3K/Akt → mTORC1, inhibition of GSK3, regulation of FOXO1.	↑ glycogen synthesis (via GSK3 inhibition & GCK), ↓ gluconeogenesis (via FOXO1 inactivation), ↑ lipogenesis (SREBP-1c via mTORC1).	[75]
Endothelial cell (vascular endothelium)	Moderate IR expression; endothelial insulin signaling is physiologically important but can be selectively impaired in insulin resistance.	PI3K → Akt → eNOS activation (NO production); MAPK/ERK mainly mediates mitogenic responses.	Vasodilation via eNOS/NO, regulation of capillary recruitment and hemodynamic–metabolic coupling; effects on vascular tone and substrate delivery.	[76,77]
Neurons / Retina (neuronal cells, photoreceptors, retinal ganglion cells)	Variable/moderate IR expression—retina and many CNS neurons express IR and are insulin-sensitive.	PI3K → Akt (survival, metabolism) and MAPK/ERK (growth/plasticity).	Neuronal survival, synaptic maintenance, metabolic support; in the retina, insulin signaling supports photoreceptor and ganglion cell function. Dysregulation linked to retinal disease.	[19,78]

From the previous table, it is notable that the retina is among the tissues with the highest IR expression. On the other hand, the retina is a tissue characterized by a high glucose demand, and its function is strongly affected by impaired insulin signaling under pathological conditions such as diabetes [78]. Therefore, the following sections describe the mechanisms and importance of systemic insulin signaling, as well as the role of insulin produced locally within the RPE for retinal function in both physiological and pathological conditions.

## 3. Insulin and Retina: State of the Art

### 3.1. Insulin Signaling Effect on Retina in Physiological and Pathological Conditions

Insulin signaling plays a central role in maintaining retinal homeostasis by regulating vascular stability, neuronal survival, and metabolic balance [79]. Insulin displays several effects on the retina since different cell types express IR, such as endothelial cells, pericytes, Müller glia, photoreceptors, retinal ganglion cells, and retinal pigment epithelium (RPE) [80,81,82,83]. In the retina, IR activation triggers intracellular phosphorylation cascades, primarily involving the PI3K/Akt and MAPK/ERK pathways. PI3K/Akt signaling promotes cell survival, inhibits apoptosis, preserves tight junction integrity, and supports metabolic functions, while MAPK/ERK regulates cellular proliferation, differentiation, and angiogenic responses [78]. Insulin also supports the regeneration of retinal ganglion cells (RGC) [84]. Daily administration of human recombinant insulin eye drops stimulates dendrite and synapse regeneration in RGCs during ocular hypertension, a major risk factor for glaucoma, the leading cause of irreversible blindness worldwide [84].

The retina has a high proclivity to damage following diabetes, and much of the pathology seen in diabetic retinopathy has been ascribed to hyperglycemia and downstream cascades activated by increased blood glucose [85]. In detail, systemic insulin deficiency or peripheral insulin resistance leads to impaired Akt phosphorylation, increased FOXO-mediated pro-apoptotic signaling, and elevated oxidative stress [86]. These molecular disruptions contribute to endothelial and pericyte apoptosis, capillary dropout, microaneurysm formation, and vascular endothelial growth factor (VEGF)-mediated pathological neovascularization [86]. Impaired insulin signaling in Müller glia further compromises neuroprotective support, heightening excitotoxicity and neuronal dysfunction [87].

Therefore, the retina may be considered an insulin-sensitive and insulin-responsive organ since several retinal cell types are responsive to insulin, and the disruption of its signaling is implicated in several retinal diseases, including retinitis pigmentosa (RP), diabetic retinopathy (DR), and glaucoma [85]. In diabetic retinas, the IR/PI3K/Akt pathway is downregulated, and retinal insulin resistance contributes to oxidative stress, inflammation, and neuronal dysfunction, thereby accelerating retinal cell degeneration [88,89]. Insulin provides trophic signals that counteract photoreceptor degeneration, a key event in RP, age-related macular degeneration, and DR [90,91]. Systemic insulin administration delays cone death in RP mouse models lacking rods [81], highlighting its neuroprotective potential.

However, retinal pathology due to an insulin-deficient signaling cannot be attributed only to local defects in the activation of PI3K/Akt and MAPK/ERK pathways. Chronic hyperglycemia, resulting from systemic insulin deficiency or peripheral insulin resistance, exerts profound vascular effects that exacerbate retinal injury [92]. High blood glucose levels promote the formation of advanced glycation end products (AGEs), oxidative stress, and inflammation, which compromise endothelial cell function and pericyte survival [93]. These vascular insults lead to capillary dropout, microaneurysm formation, and disruption of the blood-retinal barrier, further impairing nutrient and oxygen delivery to retinal neurons [94]. Elevated VEGF signaling drives pathological neovascularization, a hallmark of proliferative diabetic retinopathy, which can culminate in retinal hemorrhages, edema, and vision loss [95,96].

Therefore, the interplay between metabolic and vascular insults creates a self-reinforcing cycle: impaired insulin signaling increases oxidative stress and inflammation, while hyperglycemia-driven vascular dysfunction limits nutrient supply and clearance of toxic metabolites, amplifying neuronal and glial damage.

### 3.2. Interaction Between Photoreceptors and RPE

Among all the cell types composing the retina, the RPE and photoreceptors exhibit the strongest structural and metabolic interactions [97,98,99].

In mammals, a single RPE cell interacts with approximately 30 photoreceptors, efficiently clearing the shed photoreceptor outer segments (POS) to prevent debris accumulation and maintain retinal homeostasis [100,101]. Phagocytosis of POS follows a diurnal rhythm, peaking 1–2 h after light onset [102], and is tightly regulated by the circadian clock and metabolic signaling pathways, including those related to ATP production and lipid metabolism [103]. Disruption of this coordinated process can result in the accumulation of lipofuscin and other toxic byproducts, contributing to oxidative stress and retinal degeneration [100]. The molecular machinery of POS phagocytosis involves multiple steps. Cell surface receptors, particularly Mer Tyrosine Kinase (MerTK), recognize phosphatidylserine exposed on the POS surface, facilitating engulfment [104]. Impaired MerTK function or its aberrant shedding reduces POS internalization, compromising photoreceptor renewal [105,106]. Once internalized, POS are enclosed in phagosomes, which then fuse with lysosomes to form phagolysosomes, where macromolecules are digested [107]. Efficient resolution of phagocytosed material prevents toxic accumulation and supports RPE longevity [108,109], while digested components are recycled and supplied back to photoreceptors as metabolic substrates, such as fatty acids and retinoids [110]. Daily phagocytosis removes approximately 7–10% of POS mass, leading to full renewal every two weeks [111].

With aging or in retinal diseases such as RP, age-related macular degeneration, or diabetic retinopathy, photoreceptor turnover becomes less efficient [112]. This leads to accumulation of damaged POS, impaired metabolic exchange with the RPE, and elevated oxidative stress, ultimately causing photoreceptor apoptosis and vision loss [113]. Overall, photoreceptor turnover is a highly coordinated process involving structural renewal, metabolic support, and tight intercellular communication with the RPE [110]. Preserving this crosstalk is essential for maintaining photoreceptor health, preventing degenerative disease, and sustaining vision throughout life [114].

### 3.3. Metabolic Crosstalk Between Photoreceptors and RPE and Insulin Role in This Link

Photoreceptors are among the most metabolically active cells in the body, reflecting their continuous need to capture light, renew outer segment discs, and transmit visual information [115]. ATP is the primary energy molecule supporting these processes, generated through a combination of anaerobic glycolysis and aerobic respiration [116,117,118]. In particular, the rod aerobic metabolism is in part sustained by an ectopic oxidative phosphorylation (OxPhos) located in the disc membrane [119,120]. These extramitochondrial complexes exploit local proton gradients to generate ATP, ensuring that energy supply matches the high metabolic demand of the outer segments, which have relatively low mitochondrial density [120,121,122]. This mechanism is particularly significant under conditions of intense phototransduction or during oxidative stress, allowing photoreceptors to maintain function independently of inner segment mitochondria and ensuring photoreceptor resilience under stress and high activity [123].

Photoreceptors also rely on several metabolic pathways to meet their metabolic needs. The pentose phosphate pathway provides ribose-5-phosphate for nucleotide synthesis and NADPH for antioxidant defense, critical for neutralizing reactive oxygen species generated by light exposure and high metabolic activity [115]. Fatty acid oxidation is particularly important in cones, which contain a higher mitochondrial density and greater oxidative capacity compared to rods, supplying additional ATP for their rapid response and color discrimination functions [124]. Together, these pathways integrate with oxidative phosphorylation to ensure continuous ATP availability across both rods and cones.

In photoreceptor cells, insulin signaling is implicated in various metabolic processes, including glucose uptake and utilization [125]. Insulin receptors are present on photoreceptors, and their activation can influence the expression of genes involved in energy metabolism and antioxidant defense [125,126,127].

Disruption of insulin signaling in these cells can lead to impaired glucose metabolism, increased oxidative stress, and accelerated photoreceptor degeneration, as observed in models of DR, RP, and age-related macular degeneration [128,129]. In fact, it has been shown that systemic administration of insulin delays the death of cones in RP mouse models lacking rods [81].

RPE cells support their energy demands primarily through OxPhos rather than glycolysis, which is supported by the presence of abundant mitochondria [130]. Furthermore, RPE metabolism is sustained by their ability to metabolize various substrates, including glucose, lactate, fatty acids, and amino acids [131], adapting their metabolic pathways to satisfy the energy demand and to optimize substrate utilization [97]. The choice of substrates is regulated by their availability as well as by the crosstalk between metabolic pathways, which may produce intermediates that interfere with metabolism. For example, treatment of RPE cells with insulin-like growth factor 1, which is involved in the pathogenesis of ocular diseases, alters the energy metabolism of ARPE-19 cells by reducing oxidative OxPhos, both in terms of oxygen consumption and ATP synthesis, due to decreased activity of pyruvate kinase [132]. However, the high metabolic plasticity of RPE cells allows the activation of an adaptive response to the reduced availability of pyruvate, thereby preferentially using glutamine and fatty acids as alternative energy substrates [132]. RPE also uses glucose to produce precursors for the pentose phosphate pathway or to synthesize glycogen [133]. The majority of glucose is transported from the choroid to the subretinal space through GLUT1, which is expressed on both the apical and basolateral membranes of polarized RPE cells [134]. Also, amino acids like proline and glutamine are metabolized by RPE to produce TCA intermediates and support energy demand [135,136].

Insulin exerts multiple effects on RPE, influencing cell proliferation, metabolic activity, and protein expression. Under physiological conditions, insulin signaling in the RPE supports glucose uptake, mitochondrial oxidative metabolism, lipid turnover, protein synthesis, and the secretion of trophic factors essential for photoreceptor maintenance [78]. Therefore, when insulin signaling is sustained, the cell may favor anabolic processes and substrate uptake supporting OxPhos [78]. In addition, insulin seems able to promote RPE proliferation, potentially through the ERK1/2 pathway activation [137]. Insulin also enhances taurine uptake from RPE, which is critical for retinal function [138], and stimulates endocytosis, supporting the RPE’s role in nutrient recycling and photoreceptor maintenance [2]. At the molecular level, insulin modulates the expression of key proteins, including upregulation of the IR β subunit, and influences factors implicated in retinal diseases [2]. For instance, it reduces VEGF-A and angiotensinogen levels, while affecting TIMP-2, bFGF, MMP-2, and IGF-1 [139], highlighting its complex regulatory role in both vascular and extracellular matrix pathways. These actions are particularly relevant in the context of diabetic retinopathy, where chronic insulin resistance may alter the RPE’s responsiveness, contributing to disease progression [140].

Under oxidative stress or hypoxic conditions, RPE cells undergo several functional changes, including metabolic reprogramming characterized by enhanced glycolysis and altered substrate utilization, as well as changes in secretome composition, with increased release of VEGF, lipids, and lactate [141]. Indeed, oxidative stress damages mitochondrial components, leading to reduced ATP production and increased reactive oxygen species (ROS), further reinforcing a metabolic shift toward glycolysis. However, insulin signaling may remain active or even become hyperactivated under hyperglycemic conditions, thus promoting anabolic metabolism through the PI3K/AKT/mTORC1 axis. Integrated metabolomics reveals that prolonged mTORC1 signaling enhances glucose uptake and lipid synthesis, while also inhibiting fatty acid oxidation in RPE cells, leading to lipid accumulation and cellular metabolic stress [142]. On the other hand, oxidative stress may also impair insulin signaling [143], shifting RPE to an altered metabolic state and driving it to glycolytic metabolism or altering substrate utilization due to compromised mitochondrial respiration. Recent omics-based analyses have provided further mechanistic insight into these processes. Transcriptomic and metabolomic profiling of stressed RPE reveals upregulation of glycolytic enzymes (e.g., HK2, PFKFB3) and suppression of genes involved in mitochondrial and β-oxidation, reflecting a shift toward glycolytic metabolism [99]. Moreover, integrated metabolomics revealed enhanced glucose and lipid biosynthetic fluxes, as well as inhibited fatty acid oxidation in RPE cells with hyperactive mTORC1 signaling [142]. Single-cell RNA-seq datasets have also identified heterogeneous RPE subpopulations exhibiting differential expression of insulin-responsive and hypoxia-regulated genes, suggesting that local metabolic microenvironments shape insulin sensitivity within the RPE monolayer [144].

**Figure 2:**
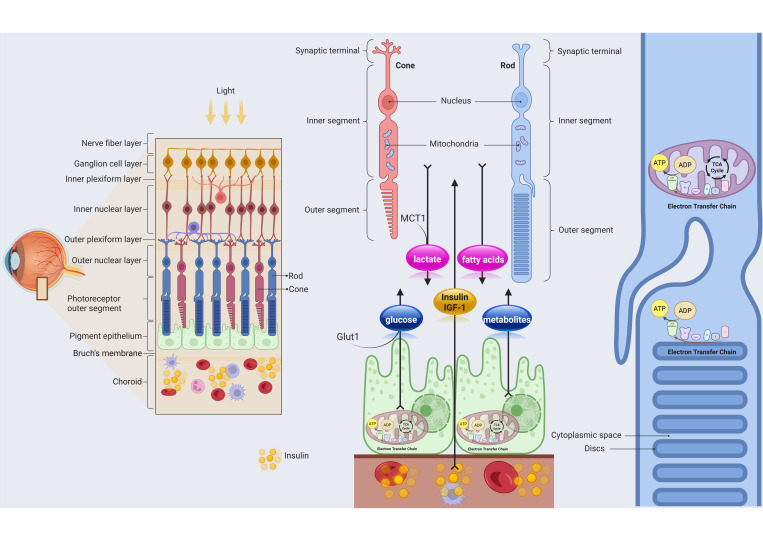
Schematic illustration of the metabolic interaction between RPE and photoreceptors. The RPE supplies glucose to rods and cones via GLUT1 transporters, while photoreceptors return metabolites such as lactate and fatty acids that fuel RPE oxidative metabolism. Insulin and IGF-1 signaling modulate this bidirectional metabolic exchange, supporting retinal homeostasis and photoreceptor function. The image was created at https://BioRender.com by A.A (accessed on 28 October 2025).

Although POS and RPE display distinct metabolic pathways to meet their specific energetic demands, metabolite exchanges between them through the interphotoreceptor matrix have also been reported [97] (Figure 2). For example, fatty acids derived from the phagocytosis of POS are converted through β-oxidation into acetyl-CoA, a key substrate sustaining OxPhos [145]. Moreover, other studies have suggested that photoreceptors, by metabolizing glucose through anaerobic glycolysis, release lactate that is taken up by the RPE via monocarboxylate transporters (MCT1) located on the apical side of the cells [115]. Once inside, lactate can either be converted into pyruvate by lactate dehydrogenase, thereby contributing to OxPhos, or be exported across the basolateral membrane via MCT3 transporters [146]. On the other hand, the same RPE provides essential nutrients, such as glucose, to the photoreceptors, which play a pivotal role in their continuous energy demands and daily renewal process [98].

In this contest, insulin signaling plays a pivotal role in the maintenance of the metabolic and functional crosstalk between POS and RPE [147] since glucose uptake from RPE represents a critical metabolic link between the blood and the POS layer and is essential to the renewal of POS, a process that ensures the continuous maintenance of retinal function and visual acuity. The POS, composed of stacked membranous discs enriched in photopigments like rhodopsin in rods and opsins in cones [148], is highly exposed to light-induced stress and oxidative damage [149]. Continuous renewal of the POS is crucial for photoreceptor health, as exposure to light not only triggers the visual cascade but also generates photo-oxidative damage to proteins and lipids [150].

In diabetes, impaired insulin signaling in the RPE contributes to retinal damage, highlighting the importance of this pathway for retinal health [151]. For example, the study by Tarchick et al. (2019), utilizing a mouse model with RPE-specific IR knockout, shows that mice exhibited reduced amplitudes of the a- and b-waves in electroretinogram recordings, indicating compromised rod photoreceptor function [2]. Interestingly, the absence of IR signaling in the RPE led to a reduction in oxidative stress markers and pro-inflammatory cytokine expression in the retina of diabetic mice [2]. These results imply that insulin signaling in the RPE contributes to the regulation of oxidative stress and inflammation, processes implicated in diabetic retinopathy. On the other hand, a recent paper of this group has demonstrated that early oxidative damage, characteristic of several retinal diseases, may originate from the photoreceptors and subsequently extend to the RPE, confirming, once more, the importance of the crosstalk between these two parts of the retina [152].

## 4. Insulin and Retina: New Perspectives

### 4.1. Local Insulin Production in RPE

Despite the role of systemic insulin on retina metabolism, a recent study conducted by Etchegaray et al. [1] demonstrate that murine RPE is a site of insulin production and release, challenging the longstanding notion that insulin is exclusively produced by pancreatic β-cells.

**Figure 3:**
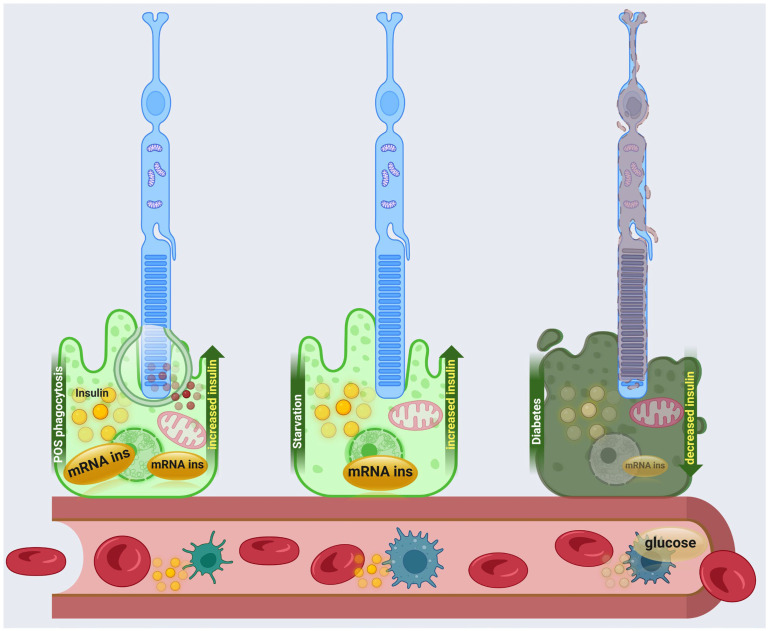
Representation of local insulin dynamics in RPE under physiological and pathological conditions. In normal conditions, POS phagocytosis stimulates insulin mRNA expression and release from the RPE, contributing to photoreceptor support. Starvation similarly enhances local insulin production as an adaptive response to metabolic stress. In contrast, diabetes markedly reduces RPE insulin synthesis, impairing glucose homeostasis and retinal function. The image was created at https://BioRender.com by A.A (accessed on 28 October 2025).

In detail, RPE express Ins2 mRNA and insulin protein, particularly in response to phagocytosis of damaged POS. Immunofluorescence analyses confirmed the presence of C-peptide, indicating active insulin secretion, in both mouse and human retinas [1]. Functional experiments revealed that deletion of phagocytic receptors significantly reduced insulin production, whereas activation of the MerTK receptor enhanced insulin synthesis, demonstrating that phagocytic activity directly regulates local insulin production in the RPE [1] (Figure 3).

Physiologically, RPE-derived insulin appears to be particularly important during fasting, when retinal phagocytosis is elevated. Loss of Ins2 specifically in RPE cells led to reduced retinal glucose uptake, impaired phototransduction, and accelerated photoreceptor degeneration in a murine model of RP [1]. These findings suggest that locally produced insulin provides a critical metabolic and neuroprotective function, supporting both glucose utilization and photoreceptor survival independently of circulating pancreatic insulin (Figure 3).

In addition, changes in local insulin synthesis occur in the diabetic retina and in response to stressors known to initiate retinal neurodegenerative processes [153]. The expression of insulin in the retina was altered with the progression of diabetes in streptozotocin-induced diabetic models in mice and donors with DR. However, it has been shown that acute stress induces the de novo insulin mRNA in isolated retinas [153], suggesting that insulin may play a role in regulating oxidative stress in diabetic retina (Figure 3).

From a translational perspective, the discovery of RPE-derived insulin opens new therapeutic avenues. Strategies to enhance or mimic local insulin production could potentially protect photoreceptors in degenerative retinal diseases and provide metabolic support during retinal stress. Moreover, targeting RPE insulin pathways may complement systemic diabetes therapies by ensuring that peripheral tissues receive adequate insulin signals independent of pancreatic function.

### 4.2. Perspective on RPE Insulin Production on Retinal Diseases

Beyond systemic insulin action, locally produced insulin by the RPE has emerged as a potentially critical protective mechanism. RPE-derived insulin could act through both paracrine and autocrine mechanisms.

From a paracrine point of view, insulin released by RPE could engage IRs on neighboring retinal cells to sustain PI3K/Akt and MAPK/ERK activity, even under conditions of systemic insulin deficiency or resistance. Mechanistically, this local insulin may preserve blood-retinal barrier integrity by regulating tight junction proteins such as occludin and claudins, reduce pathological VEGF secretion from endothelial cells and retinal macrophages, and limit vascular leakage and neovascularization. In retinal neurons, RPE insulin promotes Akt-mediated survival, inhibits FOXO-dependent apoptotic cascades, and reduces excitotoxic stress, supporting both ganglion cells and photoreceptors. Additionally, it contributes to local metabolic homeostasis by enhancing glucose uptake via GLUT1/GLUT4, regulating lipid metabolism, limiting the accumulation of AGEs and oxidative stress, and suppressing stress-induced apoptosis.

From an autocrine point of view, RPE-derived insulin may also influence the metabolic crosstalk between the RPE and POS. Although the role of insulin produced by RPE cells has not yet been fully elucidated, it is plausible that it may contribute to the metabolic homeostasis of the retina. Since the RPE is responsible for the daily phagocytosis of distal POS, insulin may help photoreceptors and RPE cells to meet the increased energy demand by upregulating glucose uptake. This hypothesis is also supported by the fact that RPE-derived insulin is regulated differently from systemic insulin. Its production is stimulated during starvation—a condition in which blood insulin levels decrease significantly, while retinal insulin levels remain unchanged [1]. This mechanism may ensure the physiological renewal of POS by sustaining the glucose demand of the RPE for the phagocytosis process, as well as maintaining sufficient glucose uptake in photoreceptors, which is essential for visual function even during starvation.

Moreover, since the RPE and POS are capable of exchanging alternative energy substrates such as β-hydroxybutyrate and lactate, and this metabolic interaction can be disrupted under diabetic or oxidative stress conditions, RPE-derived insulin may help stabilize these processes by sustaining Akt signaling, reducing oxidative stress, and promoting efficient metabolic coupling between the RPE and photoreceptors, ultimately supporting both neuronal survival and photoreceptor function.

In each case, the RPE local insulin signaling could establish a microenvironment conducive to retinal health, which may be particularly important under conditions where systemic insulin availability is insufficient or where local metabolic demands are elevated.

Clinically, leveraging RPE-derived insulin presents promising therapeutic opportunities. Enhancing local insulin production or signaling could complement systemic glycemic control or intravitreal therapies, providing direct neurovascular protection at the site of injury. Future therapeutic strategies could focus on modulating molecular pathways that regulate RPE insulin synthesis or action. For instance, enhancing MerTK signaling—a key mediator of RPE homeostasis and phagocytic function—may indirectly support insulin production and cell survival under metabolic stress. For this purpose, increasing the activity of its ligands, such as Gas6 or Protein S, preventing its cleavage by inhibiting enzymes like ADAM17, or directly stimulating the receptor through agonists or by promoting interactions with other proteins such as Tim-4, could represent valid approaches. In addition, gene therapy strategies targeting RPE-specific promoters could be employed to restore or upregulate insulin expression in degenerative or diabetic conditions. In parallel, pharmacological agents designed to potentiate local insulin receptor activity, stabilize insulin availability within the subretinal space, or deliver insulin directly to the RPE and photoreceptors may amplify neuroprotective outcomes.

By mitigating apoptosis, oxidative stress, and vascular leakage early in the disease process, extrapancreatic insulin could reduce reliance on invasive treatments such as frequent insulin injections or anti-VEGF therapy [154,155,156]. This approach may be particularly advantageous for preventing diabetic macular edema, preserving photoreceptor function, and slowing progression of diabetic retinopathy DR before irreversible damage occurs.

### 4.3. Study Limitations

However, the local insulin production by RPE opens new perspectives on retina metabolism in physiological and pathological conditions, and several critical considerations must be addressed. The regulation of RPE-derived insulin must be tightly controlled; excessive local insulin could theoretically induce localized hypoglycemia or promote aberrant vascular growth. The long-term secretory capacity and viability of RPE cells may be affected by chronic diabetic stress, oxidative damage, inflammation, and cellular senescence. Moreover, the precise pharmacokinetics, stability, and bioavailability of locally produced insulin need further investigation. Preclinical and clinical studies are essential to evaluate the efficacy, safety, and durability of therapies aimed at enhancing extrapancreatic insulin production. Despite these challenges, the concept of RPE-derived insulin provides a novel perspective on retinal self-regulation and offers a potential metabolic strategy for early intervention in retinal diseases.

### 4.4. Future Research Priorities

As future directions, further studies should be conducted in vivo to validate the synthesis of insulin by RPE, confirming its physiological relevance beyond in vitro observations. In addition, elucidating how RPE-derived insulin is regulated under conditions of metabolic stress, such as diabetes or retinal degeneration, will play a pivotal role in understanding the role of local insulin release in disease pathophysiology. In particular, studies combining single-cell transcriptomics, proteomics, and functional assays could clarify the mechanisms controlling insulin expression and secretion. Moreover, identifying how this pathway interacts with systemic insulin signaling may uncover new therapeutic targets. Finally, considering that the RPE [153] is a major contributor to insulin synthesis in co-culture systems, and the evidence that POS phagocytosis acts as a driver of insulin production in RPE cells [1], it would be interesting to investigate the effect of insulin signaling secondary to OS phagocytosis on the RPE metabolism, a relevant issue that remains unexplored. The translational potential of modulating RPE insulin production could open innovative strategies for the prevention or treatment of diabetic retinopathy and other retinal degenerative disorders.

## 5. Conclusions

Insulin signaling within the retina—both systemic and locally produced—plays a multifaceted role in maintaining vascular and neuronal integrity. RPE-derived insulin may potentially contribute to an intrinsic neurovascular-protective mechanism, possibly supporting PI3K/Akt and MAPK/ERK pathways, mitigating oxidative stress, modulating glucose and lipid metabolism, and helping to suppress pathological angiogenesis. Recognizing and harnessing this local insulin source offers promising avenues for innovative, metabolism-targeted therapies that could complement existing treatments and improve outcomes for patients with diabetic retinal disease.

## Data Availability

Not applicable.
